# Family members of older persons with multi-morbidity and their experiences of case managers in Sweden: an interpretive phenomenological approach

**DOI:** 10.5334/ijic.1538

**Published:** 2015-03-31

**Authors:** Markus Hjelm, Ann-Charlotte Holmgren, Ania Willman, Doris Bohman, Göran Holst

**Affiliations:** Department of Health, Blekinge Institute of Technology, Karlskrona, Sweden; Department of Health Sciences, Lund University, Lund, Sweden; Department of Health, Blekinge Institute of Technology, Karlskrona, Sweden; Department of Health Sciences, Lund University, Lund, Sweden; Department of Health, Blekinge Institute of Technology, Karlskrona, Sweden; Department of Care Science, Malmö University, Malmö, Sweden; Department of Health, Blekinge Institute of Technology, Karlskrona, Sweden; Department of Health, Blekinge Institute of Technology, Karlskrona, Sweden

**Keywords:** case management, integrated care, family members, interpretive phenomenology, multi-morbidity, older persons

## Abstract

**Background:**

Family members of older persons (75+) with multi-morbidity are likely to benefit from utilising case management services performed by case managers. However, research has not yet explored their experiences of case managers.

**Objectives:**

The aim of the study was to deepen the understanding of the importance of case managers to family members of older persons (75+) with multi-morbidity.

**Design:**

The study design was based on an interpretive phenomenological approach.

**Method:**

Data were collected through individual interviews with 16 family members in Sweden. The interviews were analysed by means of an interpretive phenomenological approach.

**Results:**

The findings revealed one overarching theme: “Helps to fulfil my unmet needs”, based on three sub-themes: (1) “Helps me feel secure – Experiencing a trusting relationship”, (2) “Confirms and strengthens me – Challenging my sense of being alone” and (3) “Being my personal guide – Increasing my competence”.

**Conclusion and discussion:**

The findings indicate that case managers were able to fulfil unmet needs of family members. The latter recognised the importance of case managers providing them with professional services tailored to their individual needs. The findings can contribute to the improvement of case management models not only for older persons but also for their family members.

## Introduction

Family members of older persons (75+) with multi-morbidity in Sweden are likely to benefit from case management services [1, 2] performed by case managers. As a model, case management aims to improve the coordination of health care and social services [3], thus providing integrated care [4]. However, family members’ experiences of case management and the importance of case managers for them have not yet been explored.

Since the 1990s, the amount of formal care has decreased in Sweden and today, an increasing number of older persons receive informal care from family members [5]. Informal care now substitutes for professional care in Sweden, as in many other European countries [6]. It has been estimated that around 3% of the Swedish population aged over 55 years provides care to relatives. Most of these carers are 75–84 years old and care for spouses [7].

The proportion of older persons in Europe is increasing [8]. Within this ageing population, a substantial number of older persons have multiple independent diseases, i.e. multi-morbidity [9, 10]. Older persons with multi-morbidity often have complex health and social care needs [11]. They can experience difficulties coordinating care efforts and often lack an overview of their own health and social care contacts [11, 12]. The lack of coordination and the absence of a holistic approach can result in unsatisfactory care [13], which may increase the older person's dependence on others, such as family members [12]. Consequently, the failure of the health system to meet older persons’ needs can negatively impact on their family members [5].

Family members frequently assume a great deal of responsibility for older persons’ well-being [5]. The same can be assumed to apply to family members of older persons with multi-morbidity. Furthermore, it is not uncommon for family members to feel left out when dealing with the health system [14–16]. Studies indicate that family members often consider that they have the main responsibility for the health and social care of their relatives. This responsibility may, by extension, lead to the family members suffering from various health problems [17–19]. Family members are also adversely affected if the coordination of their older relatives’ health and social care is suboptimal [5].

In Europe, the increase in informal care has led to several countries prioritising measures for relieving family members’ burden by providing professional care [6]. For instance, in Sweden, the municipalities are obliged to offer support to family members caring for persons suffering from long-term illness and/or who have disabilities [20]. However, recent investigations regarding the quality of such support services in Sweden revealed a lack of availability as well as poor quality [21], indicating that much remains to be done for these family members. There are different types of support service for family members, such as respite care and conversational support [22]. However, according to Lopez-Hartmann, Wens, Verhoeven and Remmen [22], the effects of these interventions are small and the results of studies concerning their efficacy are inconsistent. The authors suggested integrated support, tailored to the individual carers’ needs. To achieve this, it is important to coordinate the support [22]. This type of coordinated service could be provided by case managers, thus relieving some of the burden on family members.

As a model, case management aims to improve the coordination of different services, such as health and social care [3]. One of its cornerstones is thus the provision of better integrated care [4]. A proposed definition from The Case Management Society of America is as follows: “… a collaborative process of assessment, planning, facilitation, care coordination, evaluation, and advocacy for options and services to meet an individual's and family's comprehensive health needs through communication and available resources to promote quality, cost-effective outcomes” [23, p.8]. Case management has been practised in many different ways and exhibits great variety in design, addressing various aspects ranging from purely financial matters to a more individualised approach [3, 24–26]

**Figure 1.**
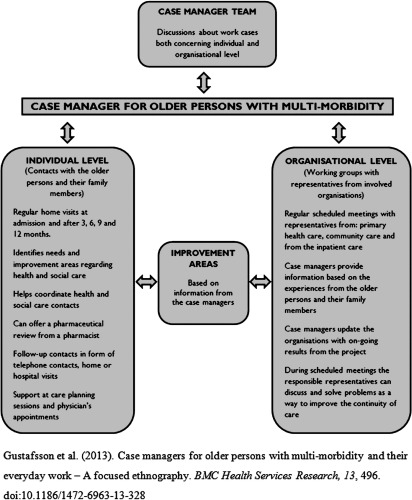
Design of the Blekings case management intervention.

Between 2011 and 2013, a case management intervention took place in Sweden [27]. Its purpose was to improve the coordination of health and social care services for older persons with multi-morbidity and their family members (see Figure 1). The case management intervention was conducted outside the existing health system by a completely new temporary organisation. From the start of the project, a total of 10 case managers from the following professional categories were involved: nurse manager, registered nurse, assistant nurse and occupational therapist. The intervention consisted of activities on two levels (see Figure 1): (1) The individual level, in which the case managers identified the different and changing needs of older persons and their family members. The case managers regularly carried out home visits, where they assessed and planned tasks based on the current concerns of the older persons and their family members. For the family members, this could include the case managers providing information regarding their older relatives’ right to effective care, guiding them to the appropriate health and social care contacts able to help them and/or their older relatives with their concerns, being available for counselling by telephone or in the course of a home visit and assisting with their older relatives’ care planning and/or physicians appointments (see Figure 1). (2) At the organisational level, the case managers identified areas where the coordination of health and social care could be improved. Family members did not actively participate at the organisational level; only at the individual level.

Besides improving the situation for older persons with multi-morbidity, case management also has the potential to improve the family members’ situation [1, 2]. However, there is a lack of knowledge about how integrated care affects family members [1]. More specifically, the experiences of case managers and the importance of case management for family members who provide help to older persons with multi-morbidity have not yet been investigated. Such knowledge could contribute valuable information for the design of case management models intended not only for older persons but also for their family members. Therefore, the aim of this study was to understand the importance of case managers to family members of older persons (75+) with multi-morbidity.

## Methods

### Design

The study design was based on Patricia Benner's interpretive phenomenology [28], which is a suitable approach for identifying similarities and differences, as well as for learning how people make sense of something [28]. This approach has been used within health and social care research [28, 29]. Interpretive phenomenology emphasises the way persons dwell in the world in terms of acting and meaning. Knowledge is sought from an ever-changing world that shapes human beings [28]. Therefore, the complexity of the specific context in which the family members reside must be taken into account. We were interested in understanding family members’ experiences of case managers and what these experiences meant for them. Hence, interpretive phenomenology was considered a suitable approach.

### Participants and study setting

The participants were selected by purposive sampling [30] in order to obtain rich narratives pertaining to the aim of the study. The recruitment therefore targeted participants who fulfilled the following criteria: had experience of being a family member of an older person (75+) with multi-morbidity and participating in the Blekinge case management intervention (see Figure 1) for at least one year.

In the present study, family members were defined as people who provide assistance to relatives, while relatives were defined as older persons (75+) with multi-morbidity in need of that assistance. This study's definition of multi-morbidity is based on that proposed by the Swedish National Board of Health and Welfare [31], which includes the following criteria: persons over 75 years, with three or more medical diagnoses from different disease groups, who have been acutely admitted to a hospital at least three times during the last 12 months.

All participants were recruited between July and the end of September 2012. Contact information for potential participants (in accordance with the study's purposive sampling criteria) was received from the case managers. These potential participants had previously been asked by the case managers if they would agree to being contacted by a researcher regarding participation in a study. ACH phoned the potential participants and informed them about the study. Afterwards, ACH sent out written information concerning the study, including a written consent form and a questionnaire for demographic data (see Table 1). ACH made a second phone call one week after sending out the information to inquire whether the potential participants were still willing to take part in the study. Subsequently, the participants decided on the time and location for the interviews. A total of 22 family members were invited, of whom 16 agreed to participate. Six family members declined, stating they had no or only limited experience of case management services.

The study was performed in the county of Blekinge in Southern Sweden. Blekinge's health system consists of primary health care, community care and inpatient care. Although the county council is the regional health care provider, the county's five municipalities are responsible for the care of older persons living in nursing homes or in their own homes [32]. The municipalities also have a responsibility to provide help to family members caring for persons who are suffering from long-term illness and/or who have disabilities. The availability of services such as respite care and conversational or financial support varies between the municipalities [5]. All the municipalities in the present study offered the following support services to family members: respite care, support groups and a coordinator for relatives who could provide information about support services to family members if requested.

### Procedure

Individual interviews with the 16 participants were conducted by ACH. The locations selected by the participants were: their own home (*n* = 13), the second author's workplace (i.e., the university; *n* = 2) and over the telephone (*n* = 1). A semi-structured interview guide [33] was used, which commenced with an open-ended question: “Can you please tell me about your experience of case managers?” This was followed by two further questions: “Can you please tell me about what you and the case managers do during your encounters?” and “Can you please tell me what case managers mean for you in your role as a family member of an older person with multi-morbidity?” Probing questions were posed to deepen the understanding of the participants’ narratives. The interviews were digitally recorded and subsequently transcribed verbatim. The transcriptions were checked for accuracy by both MH and ACH.

### Pre-understanding

In a previous study [27], MH had conducted participatory observations and interviews focusing on case managers’ everyday practices. During the observations, the author came into contact with family members and observed how the case managers interacted with them. The contextual information gained from these observations provided a pre-understanding, which was present during the data analysis. All authors had previous experience of working with older persons afflicted by complex health problems and their family members, both as registered nurses and as researchers.

### Data analysis

The interpretive phenomenological analysis was inspired by Benner [28] and involved several interrelated steps. The aim of the data analysis was to reveal the meanings in the family members’ narratives. During the entire analysis process, the interpretation moved back and forth between the parts and the whole to allow an understanding of and critical reflection on the narratives.

In the first step, all the narratives were read several times by the first author to gain a general understanding of the experiences of family members and the context of the narratives. The other authors also read the narratives, which enabled them to jointly reflect on the data. MH and ACH had several meetings comprising reflections on the narratives. In the second step, the narratives were read individually to interpret them in their own contexts, thus allowing a greater immersion in the individual perspectives. During this step, the focus was on significant episodes reported by the participants, which illuminated their experiences and meanings. During and after every reading of the interview texts, the authors wrote notes in each transcript. These interpretive notes contained comments about significant episodes found in the text. In the third step, a paradigm case (i.e., a case consisting of significant and strong episodes reflecting the family members’ experiences of case managers) was identified [28]. According to Benner, paradigm cases illustrate the practical world and may serve as a way to gain new insights about the data [28]. This study's paradigm case was intended to serve as a starting point for the results, providing the reader with an understanding of the context.

In the final step, a thematic analysis was performed, during which the transcripts of the interviews were searched for descriptions containing similarities and patterns emerged in the narratives through this iterative process. These patterns reappeared throughout the continuous readings of the narratives, leading to the identification of three themes. These themes were then reflected on by the authors, after which an overarching theme was lifted from the three themes. The authors reached consensus on the overarching theme and three sub-themes.

### Ethical considerations

This study was conducted in accordance with the ethical guidelines contained in the Declaration of Helsinki [34]. Ethical approval was applied for and granted by the Regional Ethical Review Board in Lund (Ref. No. 2012/228).

## Findings

The findings focus on family members’ experiences of case managers. One paradigm case, *Mrs K*, serves as a point of departure and consists of significant episodes interpreted and extracted from the narratives. This is followed by findings from the thematic analysis, comprising one overarching theme and three sub-themes. The overarching theme *Helps to fulfil my unmet needs* is based on the three sub-themes: (1) *Helps me feel secure – Experiencing a trusting relationship*, (2) *Confirms and strengthens me – Challenging my sense of being alone* and (3) *Being my personal guide – Increasing my competence*.

### Paradigm case: Mrs K., 63 years old, caring for her mother

At the beginning, I considered the case manager an anonymous person, but over time, a trusting relationship developed between us. At first, I was hesitant about the case manager's intentions, but realised fairly quickly that she could help us. We had been left to ourselves for quite some time without any external support. I fought many times to get my mother the support she needed and often felt alone and powerless. Suddenly, there was someone to assist us and I had not even asked for any help. It was surprising, but mainly positive.

Previously, I considered that the health care service only focused on the person in need of care. However, as a relative, I assume a great deal of responsibility for the care of my mother and I am often forgotten. I have felt abandoned, but when the case manager exhibited a genuine commitment to helping me, I felt confirmed. Now I had a contact not only for my mother but also for myself. Over time, I noticed that my mother gained a lot of confidence in the case manager. I sensed that I was no longer the only person my mother could contact if she needed assistance or was just in a complaining mood, which was a relief. At times, providing care for my mother has been very strenuous and affected my relationship with her in a negative way. Since the case manager came and provided support, it has relieved me of some of the pressure, which in turn has meant that I have had more energy for both myself and my family.

I feel that the case manager genuinely cares about the well-being of both my mother and myself. Previously, I experienced only sporadic contact when dealing with the health and social services. But the case manager has been in regular contact and also updates me on a regular basis about my mother. As she is familiar with our situation, I do not have to explain things over and over again. I feel that the case manager is on our side when dealing with the health and social services. She has the knowledge to help us contact the right health care professionals. Throughout my efforts to get the health and social service authorities to acknowledge that my mum needs help, the case manager was able to step in and confirm our situation, acting as our representative.

### Overarching theme: *Helps to fulfil my unmet needs*

The overarching theme of *Helps to fulfil my unmet needs* describes the family members’ experience of how the case managers assisted them with different services, fulfilling individual needs that were not met by the health system. The family members were not accustomed to person-centred service when dealing with the health system and felt that such service was missing. The family members had also been accustomed to sporadic contact with health care professionals. The establishment of a trusting relationship with the case managers made them feel more secure in their role of providing assistance to relatives. This revealed how the family members’ sense of being alone was changed by the case managers. Previously, the family members had experienced how professional services were directed towards their older relative and, as a consequence, they often felt abandoned. Their relationship with the case managers gradually confirmed and strengthened them in their role of providing help to older persons with multi-morbidity. Their interaction with the case managers constituted a new type of contact, in which the case managers acted as their personal guide by providing individualised information about the health system and by facilitating reflection on their personal concerns. The case managers were also proactive, trying to anticipate where to solicit help before problems became more complex. Having contact with the case managers deepened the family members’ own competences and knowledge, enabling them to better assist their older relatives.

#### Sub-theme: *Helps me feel secure – Experiencing a trusting relationship*

Contact with the case managers contributed to a sense of security among the family members, which increased as the contact developed into a more trusting relationship. For this trusting relationship to mature, both the participants themselves and their older relatives had to have confidence in the case managers’ good intentions to assist them. The participants felt relieved when the relationship with the case managers developed, as their relatives could contact the case managers (rather than the participants) for assistance. One participant stated:

*“It's a support for us brothers and sisters as well because we feel that mum trusts her and that it functions well. Mum can phone her as well as us when she needs to complain a little and when she finds it difficult, then she can get a bit of a push in the right direction.”* (Participant 2)

One participant expressed her desire to stay in contact with the case manager as a guarantee for the future in case she needed help, saying, “So it is insurance for the future”. Another participant indicated that although she currently did not need any assistance from the case manager, she felt secure knowing that there was someone she could turn to if any problems arose in the future. As she had already established contact, she felt able to make contact in the future:

*“And…yes…I suppose I felt that I didn't need her all that much, but I was very happy that she was there and…knowing that I could turn to her without any difficulty.”* (Participant 5)

The participants also considered that their case managers served as a conversational support for their older relatives. In other words, there was someone other than themselves who took the time to sit down and listen to their relatives, which was a relief. However, some of the narratives revealed that despite relieving their strain, contact with the case managers also made them uncomfortable. This feeling originated in the assumption that their older relatives utilised the case managers as new contacts to whom they could complain. One participant reflected on the difference between the older relatives complaining to them and to the case manager:

*“She probably has to listen to many of the complaints that we've previously heard; well, it sounds awful, but the complaining…it's a relief for us that she gets to hear some of it, but as it's her profession, she can handle it in a different way. For us relatives it goes straight to the heart.”* (Participant 2)

The narratives revealed that the participants sometimes recognised the case managers as having the role of a “relative” within their families. This role meant that the case managers took over activities that the participants were used to doing by themselves when assisting their older relatives. The participants felt secure in the knowledge that there was someone who could assist their older relatives if they were not available. One participant stated:

*“She was there and raised these questions because I couldn't get time off work to attend, so she assumed my role as a relative, so to speak. It was fantastic to know that somebody was there to represent me or help with the things I usually do.”* (Participant 3)

The participants experienced that their contacts with different representatives of the health system were mostly of a temporary nature and recognised the need for more regular contact. The case managers had regular contact with the family members and their older relatives, both through telephone communication and home visits. Some of the family members were surprised when the case managers phoned and asked if they were in need of any assistance. This was a completely new situation for them, as they were used to having to contact various health care representatives by themselves. The family members emphasised the importance of not having to explain their situation over and over again. They felt secure in the knowledge that the case managers were familiar with their current concerns and situation, which relieved some of their strain. One participant stated:

*“…and she helped me towards the end when dad was very ill. When I didn't have enough strength she made phone calls and arranged care planning meetings, in which she took part. As she had met dad several times and knew him a little, she could…when I was not able to go, she could help, which was a huge relief and gave me a sense of security.”* (Participant 3)

#### Sub-theme: *Confirms and strengthens me – Challenging my sense of being alone*

The narratives revealed how the participants often felt alone while providing help to their older relatives. This feeling of being alone was increased by having to struggle to obtain health and social care for their older relatives. Additionally, they did not feel understood or appreciated for their efforts as carers by many health care professionals. However, these feelings changed when the case managers came to their assistance. The participants felt that their value and efforts as carers were confirmed by the case managers in a way that they had not experienced before. This confirmation strengthened them and challenged their sense of being alone. One participant stated:

*“Many care planning meetings have taken place at the hospital over the years, but the first time the case manager took part, I experienced it as an unbelievably big support because I often felt really alone sitting there trying to get the care manager and all those involved to understand that it cannot go on any longer and that he isn't aware of it himself. Then, the case manager participated and supported us by saying that it's exactly as the children have said; it cannot be allowed to continue.”* (Participant 1)

The narratives showed that many of the participants felt that they were the only caregiver for their older relative. As the case managers came into their lives and assumed some of the responsibilities for the care situation, they started to feel less lonely. One participant commented:

*“For me it was…it was just as if…I felt that it wasn't just me who was responsible there was somebody else, a professional.”* (Participant 16)

The participants acknowledged the case managers as representing their needs as family members. The case managers were perceived as being there to address their concerns and provide help for them and their older relatives without any hidden agenda. As the case managers did not represent any of the health or social care organisations linked to the care of their older relatives, the participants felt that they could trust in the case managers’ good intentions. One participant stated:

*“I experienced a sense of security that, as the child of these two old people, I had somebody I could turn to and talk to, who wasn't employed there, but who was independent of the place where they lived. Because…very often, you feel utterly helpless, when you have nobody to turn to for help.”* (Participant 7)

Another participant expressed that family members’ needs are often forgotten in today's health system, creating a sense of isolation. However, the presence of the case manager changed the participants’ feelings of being alone:

*“The focus is on the person who is ill or needs help and the family members are forgotten. And now that family members are expected to take care of so much more than previously, it is essential that they obtain the support to do it and don't feel alone or abandoned. We have felt like this for many years, thus when the case manager suddenly entered the scene it felt completely different, having somebody to turn to.”* (Participant 1)

#### Sub-theme: *Being my personal guide – Increasing my competence*

The narratives indicated that the family members experienced the case managers as being their personal guide and navigating them through the health system. They recognised the importance of benefitting from the case managers’ professional knowledge. Such professional knowledge regarding how to efficiently navigate through the health system was, in some instances, previously unknown to the participants and increased their competence. The case managers were seen as an intermediary between the family members and the health system. Through the case managers, family members could easily receive information about where and how they could acquire the best possible care for their older relatives. The case managers were seen to work pro-actively, trying to anticipate where help could be obtained before the problem escalated. Due to the fact that the family members already had personal contact with the case managers, they felt that they could receive more individualised information regarding their concerns. Access to the services of the case managers helped the family members to increase their own knowledge of the health system. One participant expressed:

*“An intermediary, you could say, who knew the paths through the health system…opportunities to obtain the best possible help for my parents.”* (Participant 13)

Besides drawing on case managers’ professional knowledge about how to navigate the health system, some of the participants expressed that they utilised the case managers as contacts with whom to discuss and reflect on their current concerns. This enabled the family members to gain different and sometimes even unknown perspectives regarding the care of their relatives:

*“Yes but…there were…two angles, you see…I mean, she looked at my parents from another angle. Because she might be aware of help that I don't know about.”* (Participant 13)

The narratives showed that the participants believed they could contact the case managers for guidance about various concerns and that they did not feel uncomfortable about doing so. They were certain that the case managers would never disregard their concerns, but always try to help them. This contrasted with their feelings about seeking help from the health system. For some of the participants, this change was experienced as a major development. The participants were no longer afraid of being misunderstood or mistreated and thus not receiving the necessary help. One participant appreciated no longer having to always contact the health and social care services when she needed advice.

*“And I think I found it good from the start that there was somebody with whom you could exchange views instead of going directly to the hospital and getting caught up in the care system. Because you don't get answers to everything there. Well, they say…however well-meaning they may be, they may not be able to provide answers to everything.”* (Participant 14)

## Discussion

The family members were in general agreement that the services of the case managers were important to them as well as to their older relatives. Their interaction with the case managers helped to fulfil their unmet needs. They emphasised the significance of having a regular contact person who was knowledgeable about the health system and worked proactively to support them. This type of regular contact was something they had previously missed. However, it is not common for family members to be included as active participants in case management. Based on our findings, we argue that family members of older persons with multi-morbidity should be included as part of the caseload within case management interventions, thus receiving greater attention for their needs.

Case management plays a significant role in the provision of integrated care [35], and represents one way of trying to intervene in a highly specialised, complex health system which risks leading to fragmented care for its users [1, 2, 36]. Presently, the evidence recommending case management interventions targeting older persons is limited. Previous studies within the field have primarily focused on the effects of health care usage and health care costs [1, 2, 36]. These studies display a discrepancy in their results, indicating both positive outcomes and no effect at all following the intervention [1, 2, 36]. Difficulties in establishing evidence for case management could arise because many of the interventions involve different elements, are conducted in different contexts or measure different outcomes [1, 2, 36]. Furthermore, case management interventions usually do not include the assistance of the family members’ needs as an element of the intervention, thus making it hard to establish any evidence regarding the intervention's effectiveness in addressing such needs. Nevertheless, our findings illustrate that the case managers were perceived to be of great importance in fulfilling family members’ unmet needs, even when they had not been addressed by the present health system. Based on our findings, we recommend that future studies consider the inclusion of outcomes in the study design, thereby measuring the extent to which the needs of the family members have been affected by the intervention. This would help to further investigate the evidence base for case management interventions.

Our findings revealed that case managers changed the family members’ sense of being alone by paying attention to them. The family members’ unmet needs included wanting to be confirmed and have someone take an interest in them as individuals. Some even felt forgotten by the health system: *“The focus is on the person who is ill or needs help and the family members are forgotten”*. Based on our findings, one way to address this seems to be by providing them with individualised case management services, further highlighting the need to apply a person-centred approach within case management, which is also emphasised by Goodwin [35]. Correspondingly, previous studies [37, 38] acknowledge the significance of focusing on family members’ individual needs [37, 38]. So doing may affect the family members’ perceived quality of life in a positive way [38].

Another important finding was the significance of a trusting relationship between the family members and the case managers. Previous research [37] indicates the importance of trust between health professionals and family members. To build this trust the family members must be given sufficient time and space to express their needs, concerns and feelings to the health professionals [37]. With regard to our findings, we recommend that there should be strategies specifically aimed at facilitating trust at the start of case management interventions. Additionally, for this trusting relationship to evolve, it was important that the case managers did not represent any of the existing health or social care organisations linked to the care of the family members’ older relatives. Kane [39] suggested that for professional guidance to be effective, it should be provided by an informed but disinterested facilitator [39]. This view corresponds with our findings, as we found it beneficial that case managers were not affiliated with any organisation directly linked to the care of the family members’ older relatives.

Our findings indicated the importance of regular contact, providing easy access to professional knowledge of the health system and its services. The family members described the major difficulty of seeking contacts with health system representatives. In contrast, seeking contact with case managers was viewed as easy and positive. Previous research [40] highlights the importance of easy access to professional advice, especially among family members providing care for their older relatives [40]. Robben, van Kempen, Heinen, Zuidema, Olde Rikkert, Schers et al. [41] found that at times, requesting professional advice could be difficult for family members, who might lack the assertiveness to ask for such advice. Furthermore, health professionals’ behaviour during information exchanges could also be important for whether family members are confident enough to ask for professional advice [41]. We argue that when designing case management interventions, aspects such as providing easy access to professional advice through both telephone contacts and home visits, as well as specifically assigning case managers to act as individual contacts for both the family member and her/his older relative, could be beneficial for building trust between the two parties, thus providing a good foundation for an exchange of information.

Another important reflection raised by our findings is: Perhaps it should not only be the responsibility of the family members to seek assistance, but we as health professionals could also be the ones to initiate contact with family members and thus offer assistance. According to Oeseburg, Wynia, Midde and Reijneveld [36], modern day health systems are becoming more fragmented and interventions such as case management may prove important for bridging the gaps [36]. In view of the fragmentation of our health system, we might have to consider that there is a need to provide case management services not only for older persons with multi-morbidity but also for their family members.

### Methodological considerations

To enhance the interrater reliability of the findings, all the authors were involved in the review of the narratives. During the entire data analysis process, the authors made continuous critical reflections on both the shared meanings and the individual narratives. One point of discussion was that only two male family members were included in our sample. This uneven gender distribution may have affected the findings in that important gender perspectives might have been missed. However, it is not uncommon for women to be the primary family members providing help to their older relatives [7, 42]. Half of the family members had a university degree (see Table 1), which might have influenced the results as highly educated people tend to utilise health and social care services more extensively [42].

Another methodological consideration is that of the recruitment process. The case managers provided a list of potential participants based on the purposive sampling criteria. Before being entered into this list the participants had been asked by the case managers if a researcher could contact them regarding participation in a study. To ensure that the participants’ consent was given voluntarily, they were extensively informed about the purpose of the study and that their decision whether or not to participate would not affect their relationship with the case managers in any way. An additional consideration related to the recruitment process is that six potential participants declined participation, stating they had no or only limited experience of case management services. These family members could possibly have contributed different perspectives to those who agreed to participate in the study.

Another way to deepen the data could have been by conducting repeated interviews over time [43] in a prospective longitudinal design. This approach would have enabled us to pose clarifying questions, thus facilitating our interpretation of the narratives. As the first author did not conduct the interviews, additional focus was placed on attentively listening to the recorded interviews. Furthermore, the first author discussed the interviews thoroughly with the second author, who conducted them. In an earlier study [27], the first author carried out an ethnographic study of case managers’ everyday practice, which provided important contextual information that facilitated understanding of the family members’ experiences.

## Conclusions

The findings from the data suggest that case managers were able to fulfil previously unmet needs among the family members, who recognised the importance of having case managers to provide them with professional services. The family members experienced a trusting relationship with the case managers, which confirmed and strengthened them in their role of providing help to older relatives with multi-morbidity. Our findings can contribute to the improvement of case management models intended not only for older persons, but also for their family members.

## Figures and Tables

**Table 1.**
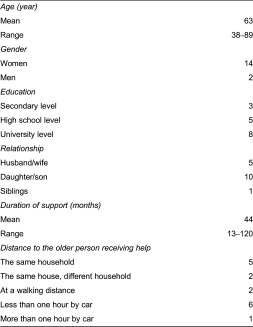
Demographic characteristics of the family members (*n* = 16)
